# *Loci* Identification of a N-acyl Homoserine Lactone Type Quorum Sensing System and a New LysR-type Transcriptional Regulator Associated with Antimicrobial Activity and Swarming in *Burkholderia Gladioli* UAPS07070

**DOI:** 10.1515/biol-2019-0019

**Published:** 2019-06-24

**Authors:** E. Seynos-García, M. Castañeda-Lucio, J. Muñoz-Rojas, L. López-Pliego, M. Villalobos, R. Bustillos-Cristales, L. E. Fuentes-Ramírez

**Affiliations:** 1 Lab. Ecología Molecular Microbiana, Centro de Investigaciones en Ciencias Microbiológicas, Instituto de Ciencias, Benemérita Universidad Autónoma de Puebla. Edif. IC11, Ciudad Universitaria, CP 72570, Puebla, Puebla, México; 2 Centro de Investigación en Biotecnología Aplicada-Instituto Politécnico Nacional, Carretera Estatal Sta Inés Tecuexcomac‑Tepetitla, km. 1.5, C.P: 90700 Tepetitla de Lárdizabal, Tlaxcala,Mexico

**Keywords:** AHL, Transposon Himar1, Antibiosis, Quorum Sensing, Toxoflavin

## Abstract

A random transposition mutant library of *B. gladioli* UAPS07070 was analyzed for searching mutants with impaired microbial antagonism. Three derivates showed diminished antimicrobial activity against a sensitive strain. The mutated loci showed high similarity to the quorum sensing genes of the AHL-synthase and its regulator. Another mutant was affected in a gene coding for a LysrR-type transcriptional regulator. The production of toxoflavin, the most well known antimicrobial-molecule and a major virulence factor of plant-pathogenic *B. glumae* and *B. gladioli* was explored. The absence of a yellowish pigment related to toxoflavin and the undetectable transcription of *toxA* in the mutants indicated the participation of the QS system and of the LysR-type transcriptional regulator in the regulation of toxoflavin. Additionally, those genes were found to be related to the swarming phenotype. Lettuce inoculated with the AHL synthase and the lysR mutants showed less severe symptoms. We present evidence of the participation of both, the quorum sensing and for the first time, of a LysR-type transcriptional regulator in antibiosis and swarming phenotype in a strain of *B. gladioli*

## 1 Introduction

Most microorganisms depend on quorum sensing (QS) as a means for detecting and facing environmental conditions. Individual cells release signaling molecules that are detected by the rest of the population and other bacterial communities living in their microhabitats. They function by making the bacterial cells aware of the density of their own population and of other species as well. Besides this, cells receive information on mass transfer in their microhabitat. These signaling molecules also play a critical role in the activation or silencing of specific metabolic pathways in order to cope with current environmental conditions [1]. The physiological responses regulated trough QS are diverse; including bioluminescence, conjugation, virulence, antibiotic synthesis, extracellular protease activity, response genes to oxidative-conditions, flagellar morphogenesis, swarming motility, root nodulation, antibiotic resistance, biofilm formation, reduction of oxidative stress and glutamate uptake [2, 3, 4, 5, 6, 7, 8, 9, 10, 11, 12].

The LysR family of transcriptional regulators (LTTR) is involved in the local or global regulation of genes associated to virulence, metabolism, quorum sensing and motility. LysR-type proteins act as transcriptional activators or repressors of genes, including negative self-regulation. There are two principal domains of LysR-type proteins, an N-terminal domain of DNA-binding and a C-terminal domain of co-inducer-binding which appears to be essential for its proper functioning. The co-inducer is often an intermediate or a metabolic product of the pathway, and its binding generates a feedback loop [review 13].

A recent re-classification of *Burkholderia* left the animal and most of the plant-pathogenic organisms in the same genus, including *B. gladioli*. The remaining species were included in the *Paraburkholderia*, *Caballeronia* and *Robbsia* genera [14, 15, 16]. Several species in *Burkholderia* can degrade pollutants, while others can produce various extracellular products such as siderophores, antimicrobials, toxins and extracellular enzymes [17, 18, 19]. In addition, antimicrobial activity has been detected for a number of distinct species of *Burkholderia*, and include, *B. bryophila*, *B. megapolitana*, some members of the *B. cepacia* complex*, B. multivorans, B. tropica*, *B. thailandensis* and *B. contaminans* [20, 21, 22, 23, 24, 25, 26, 27]. Several *B. gladioli* strains show pathogenicity towards immunocompromised humans, plants, animals and inhibitory activity against fungi and other bacteria [28, 29, 30, 31, 32, 33, 34, 35, 36, 37, 38, 39]. Genotypes of *B. gladioli* can also synthesize several molecules with different antagonistic mechanisms. Those antimicrobial molecules include: i, Bongkrekic acid, an antagonistic substance against fungi [40]. This substance is a potent toxin in humans, there have been a number of life threatening and sometimes lethal intoxications following the consumption of a native Indonesian fermented food `Tempe bongkrek´, contaminated with *B. gladioli* pv. *cocovenenans* [41]; ii, Enaxyloxin, an antibiotic produced when *B. gladioli* is exposed to *Rhizopus microspores* under O_2_ limitation [38]; iii, Toxoflavin, synthesized by *B. glumae* and *Pseudomonas protegens* Pf-5, besides *B. gladioli*. Toxoflavin is active against a wide range of bacteria and fungi [42, 43, 44, 45, 46], it functions by transferring electrons between NADH and oxygen producing hydrogen peroxide [42]; iv, Gladiolin, a broad spectrum antibiotic of the macrolide antibiotic family produced by *B. gladioli* BCC0238 [47]; and v, a cyclic peptolide antibiotic with activity against gram-positive bacteria [48].

The global Quorum-sensing mechanism regulates toxoflavin biosynthesis in *B. glumae* BGR1 and in *B. gladioli* BSR3 [49, 50, 51, 52, 53]. Furthermore, QS regulates a number of activities, this includes the synthesis of antimicrobial metabolites in other *Burkholderia* species. In *B. thailandensis*, the synthesis of an antibiotic of the bactobolin/actinobolin family [25,26]. The pyrrolnitrin production in species of the *B. cepacia* complex [21]. In *B. ambifaria*, the transcription of genes that potentially participate in the synthesis of pyrrolnitrin, enacyloxins, and occidiofungins [54].

Swarming is a bacterial collective movement over a surface and is used for colonization [55, 56]. The Acyl-Homoserine Lactone-type Quorum-Sensing system regulates swarming in different bacteria including *B. cepacia* H111, *B. glumae* BGR1, and other species [8,52,57,58].

*B. gladioli* UAPS07070 is a bacterium isolated from pineapples. It displays a wide range of antimicrobial activity against different microorganisms including Proteobacteria, Firmicutes and fungi [37]. The antagonistic mechanisms shown by that strain has not been characterized yet. Our aim was to identify and characterize *loci* associated with the antibiosis and swarming phenotypes exhibited by *B. gladioli* UAPS07070.

## 2 Methods

### 2.1 Bacterial strains, plasmids and culture conditions

The plasmids and strains of this study are shown in Table 1. *B. gladioli* UAPS07070 and the mutants were grown on Luria Bertani (LB) agar plates or liquid medium at 30oC or 37oC, depending on the experiment. *E. coli* DH5α was grown on LB plates or in LB broth at 37oC. Kanamycin (Km) was supplemented when required (80 μg/ml).

**Table 1:** Bacterial strains and plasmids.

Strain or Plasmid	Relevant Characteristic(s)	Reference or source
*Burkholderia gladioli*		
UAPS07070	Wild type strain isolated from pineapple in Mexico; Microbial antagonistic activity^+^	[39]
BG1232	UAPS07070 *tofI*::*Himar1* Diminished antagonistic activity	This study
BG87	UAPS07070 *tofR*::*Himar1* Diminished antagonistic activity	This study
BG1232-pAHL-7	BG1232 transformed with pAHL-7; positive for antagonistic activity	This study
BG1232-pBBR1MCS-5	BG1232 carrying pBBR1MCS-5	This study
BG87-pQSR-15	BG87 transformed with pQSR-15; positive for antagonistic activity	This study
BG87-pBBR1MCS-5	BG87 carrying pBBR1MCS-5	This study
BG79	UAPS07070 *lysR*::*Himar1*; Antimicrobial activity	This study
*Acinetobacter sp*		
UAPS0169	Wild type strain isolated from *Neobuxbaumia macrocephala*; Sensitive strain	[87]
*E. coli*		
DH5α	*supE44 ΔlacU*169 (φ80lacZΔM15) hsdR17 thi-1 relA1 recA1	[88]
DH5α-pAHL-7	DH5α with pAHL-7	This study
DH5α-pBBR1MCS-5	DH5α with pBBR1MCS-5	This study
DH5α-pQSR-15	DH5α with pQSR-15	This study
*Chromobacterium violaceum*		
CV026	Biosensor of Quorum Sensing	[64]
Plasmid		
p1232	Recircularized construct of approximately 8-kb of chromosomal DNA of BG1232 digested with *Not*I, containg flanking regions upstream and downstream to *tofI*::*Himar1*; R6K *ori*, Km^R^	This study
p87-2	Recircularized construct of approximately 9-kb of chromosomal DNA of BG87 digested with *Not*I, contain flanking region to *tofR*::*Himar1*; R6K *ori*, Km^R^	This study
pBBR1MCS-5	A broad host range cloning vector, RK2 *ori*, *lacZ*α, Gm^R^	[70]
pAHL-7	pBBR1MCS-5 containing a 1242-pb fragment of *tofI* plus flanking regions, obtained with a *Sal*I fragment of plasmid p87-2; Gm^R^	This study
pQSR-15	pBBR1MCS-5 containing a -pb fragment of *tofR* plus flanking regions, obtained with a *Sal*I-*Xho*I fragment of plasmid p1232; Gm^R^	This study
pHBurk3	Plasmid containing *Himar1* (with ori*R6K*, FRT-*nptII-*FRT gene, inverted repeats) and *tnp* gene, fragment containing ColE1 *ori*, *oriT*, and *ori1600*-*rep*(*TsBt*), the *nptII* facing toward *tnp*	[59]

### 2.2 Mutagenesis with *Himar1*

A randomly mutated library of *B. gladioli* UAPS07070 was obtained containing the transposon *Himar1*, an efficient genetic tool for *B. gladioli* and other *Burkholderia* species [59,60]. Additionally, *Himar1* possesses an origin or replication that allows for the recovery of sequences next to the transposon insertion even without cloning them into another vector. The suicide plasmid pHBurk3 was introduced into *B. gladioli* UAPS07070 by electroporation [61]. Electrocompetent cells were obtained as follows; cells were inoculated in 1 ml of LB broth and incubated at 30oC under agitation for 24 h. The bacterial cells were washed twice with 300 mM sucrose at room temperature and the pellet was resuspended in 100 μl of the same solution. The conditions of the electrical pulse were 25 μF; 200 Ω; 2.5 kV on a Bio-Rad GenePulserXcell. The transformants were selected in plates of LB kanamycin (80 μg/ml), incubated at 37oC until isolated colonies appeared. The mutants were preserved at -70oC in 50% glycerol (v/v).

### 2.3 Screening of mutants with reduced antimicrobial activity

Inhibition-impaired mutants against the sensitive bacterium *Acinetobacter* sp. UAPS0169 were initially screened with a multiple antagonism assay as described below. The mutants were grown with agitation in LB broth at 30oC for 24 h. 20 μl of this culture was plated on LB agar and incubated for 24 h at 30oC. Thereafter, 20 μl of culture of the sensitive strain in stationary phase was placed in the vicinity of the antagonistic strain growth without touching it. The cells were further incubated at 30oC for 24 h. Candidates for loss of antagonism were detected by the growth of the sensitive strain into the radius of one centimeter starting in the edge of the cell mass of the mutant. Double layer assay [Modified from 62] corroborated the phenotype. All antagonistic strains were grown in LB broth at 30oC for 24 h to reach stationary phase. The cultures were adjusted to an OD_620_ of 0.05 in LB broth, 6 μl was inoculated onto solid LB agar plates and incubated for 24 h at 30oC. Subsequently, the bacterial growth was removed with a sterile glass slide, and the remaining cells were inactivated by exposure to chloroform vapors for 1 h. The plates were left opened in a laminar flow cabinet until the residual chloroform had been evaporated. *Acinetobacter* sp. UAPS0169 was grown and its density was adjusted in a similar manner to the mutants. 200 μl of the adjusted *Acinetobacter* culture was spread onto the LB agar plates where the mutant clone had been previously grown. The plates were incubated for 24 h at 30oC and inhibition halos were measured. For statistical analysis, the double layer assay was performed with twelve repeats for each mutant clone. The statistical significance was tested with ANOVA (p < 0.05).

### 2.4 Identification of transposition location

The transposon-chromosomal junction in clones with decreased antagonistic activity was rescued according to the methodology described by Rholl et al. [59]. Briefly, DNA was extracted and purified with Wizard Genomic DNA Purification kit (Promega Co). The DNA was digested with *Not*I (Thermo Fisher Scientific) and purified with the High Pure PCR Cleanup Micro Kit (Roche), and self-ligated with the Rapid DNA ligation kit (Roche). The DNA was transformed into *E. coli* DH5α, and plated on LB agar plates containing Km (30 μg/ml) at 37oC. Plasmid extraction was conducted with the Pure Yield Plasmid Miniprep System kit (Roche). All kits were used following manufacturer’s instructions. The mutated sites were sequenced with the transposon specific primers 1670 (5`-TCGGGTATCGCTCTTGAAGGG-3`) and 1829 (5`-GCATTTAATACTAGCGACGCC-3`). Blastx (http://blast.ncbi.nlm.nih.gov/Blast.cgi?PROGRAM=blastx&PAGE_TYPE=BlastSearch&LINK_LOC=blasthome) was used to compare the sequences. Multiple sequence alignment with ClustalW was used to calculate the percentage of identity of the translated orfs [63]. The DNA sequence was deposited in NCBI GenBank with the acc. numbers KP123645 and MF325937. The orfs were searched with the Interproscan platform (http://www.ebi.ac.uk/interpro/search/sequence-search) to identify conserved domains in TofI, TofR and LysR.

### 2.5 Acyl homoserine lactone assay

The secretion of acyl homoserine lactones was detected with the quorum sensing indicator strain *C. violaceum* CV026 [64] which exhibits a purple pigmentation in the presence of AHLs. Cells were grown in LB broth to the stationary phase, a layer of *C. violaceum* CV026 (approximately 200 μl) was distributed over a PSUC plate (4.2 mM succinic acid, 1% casein peptone and 1.6% bacteriological agar). The plates were left opened until the suspension had dried up. 10 μl of the bacterial suspension of the tested cells was placed onto each plate and incubated for 24 h at 30oC. A positive reaction was indicated by the presence of a violet halo around the colony (violacein production).

### 2.6 Swarming assay

Bacterial cultures were grown for 24 h at 30oC in LB broth. One ml of bacterial culture, with an OD_620_ of 0.01, was washed twice with sterile LB broth. A volume of 1 μl of bacterial cells was placed in the center of an LB plate and incubated either at 30oC or 37oC for 48 h. There were a total of five replicates for each sample. A dendritic pattern formation was regarded as positive for swarming.

### 2.7 Toxoflavin quantification

The toxoflavin production was quantified in solid medium. *B. gladioli* UAPS07070 or derivate mutants were grown to stationary phase in LB broth with an OD_620_ of 0.05. 200 μl of suspension was spread over LB plates and incubated at 30oC for 24 h. The cell mass was removed mechanically with a glass slide and the agar surface was cleaned with a swab soaked with 70% ethanol to remove the remaining cells. An agar fragment (500 mg) where the cells had been grown, was sliced up and resuspended in 500 ml of chloroform. The chloroform fraction was transferred to fresh polypropylene tubes and the solvent was evaporated. The residue was dissolved in 80% methanol and the absorbance was measured at 393 nm [65]. The absorbance lectures were compared with ANOVA (p < 0.05) using six replicates.

### 2.8 End-point RT-PCR analysis of *toxA* gene

The transcription of *toxA* gene by *B. gladioli* UAPS07070, by QS and by LysR mutants were analyzed by RT-PCR. Bacterial cells were grown at 30oC in LB broth until reaching the stationary phase with an OD_620_ = 0.05. 30 μl of this bacterial suspension was plated on LB plates and incubated for 24 h at 30oC. The cells were recovered in 1 ml of RNase free water and centrifuged at 4oC and 8,000 x g for 3 min. The simple phenol method [66] was used for total RNA extraction and were as follows: 100 μl of RNase free water was added to the pellet and the cells were vortexed for 3 min, 100 μl of acid phenol-chloroform (1:1) was added and the tube was vortexed for 1 min. Following this, tubes were incubated at 70oC for 30 min, vortexing each for 5 min. The sample was centrifuged at 12,000 x g for 10 min, 100 μl of aqueous phase was transferred to a clean tube and 200 μl of isopropanol was added. The tube was vortexed for 3 min and centrifuged at 12,000 x g for 10 min, the RNA pellet was washed twice with 200 μl of 70% ethanol and centrifuged at 12,000 x g for 5 min. The pellet was air dried and RNA was solubilized in 25 μl of RNase free water. The integrity of the RNA was qualitatively evaluated in 1% agarose gel electrophoresis. The concentration and purity were measured in a NanoDrop spectrophotometer (Thermo Scientific). RNA (4 mg) was treated with DNaseI (Thermo Fisher Scientific) according to manufacturer’s instructions. For synthesis of cDNA, 2 mg of DNA-free RNA was used. cDNA was generated with the Transcriptor High Fidelity cDNA Synthesis Kit (Roche) according to manufacturer’s instructions. The *toxA* gene expression was evaluated by PCR amplification of a fragment of 181 bp using the oligonucleotides RTP3 (5`-GTT CAG CTT CTA CCG CTG GA-3`) [45] and TOXA2 (5`-TCA AGG CTT GCA GAC CAG-3`) [48]. A 412 bp fragment of 16S rDNA gene was amplified as control of constitutive expression with the oligonucleotides Fwd (5`-GTG CCA GCM GCC GCG GTA ATA C-3`) and Rev (5`-CCG TCA ATT CCT TTG AGT TT-3`) [67]. 1 μl of cDNA was used for amplification of both, *toxA* and 16S rDNA with the following conditions: 96oC for 2 min, 30 cycles at 96oC for 1 min, 50oC for 1 min for *toxA*; and, for 16 rDNA, 58oC for 1 min and a final extension at 72oC for 30 s.

### 2.9 Assay for virulence in onion and lettuce

*B. gladioli* UAPS07070, BG1232, BG87 and BG79 were grown until reaching the stationary phase at 30oC in Luria-Bertani broth. Cultures were washed twice with sterile culture medium and adjusted to OD_620_ = 0.05 (ca. 10^7^ cfu/ ml). For the onion assay a modification of the method of Jacobs *et al*. [68] was used. Fragments of ‘Yellow Globe’ onion bulbs (ca. 10 cm^2^) were inoculated with 5 ml of bacterial suspension inside of the inner surface from a wound made with a micropipette tip. The onion bulbs were incubated in a wet chamber at 30oC for 48 h and virulence was demonstrated by tissue maceration. For the assays in lettuce plants three bright leaves of four weeks old plants were inoculated in the midribs with 10^3^ washed CFU in 10 ml. The control was inoculated with 10 ml of 10 mM MgSO_4_. The plant were maintained in humid conditions and the appearance was observed daily for 5 days [Modified from 69].

### 2.10 Complementation strategy

The AHL synthase defective mutant BG1232 was complemented in trans with the plasmid pAHL-7 (Table 1 and Fig. S1) harboring 615 bases of *tofI* plus 386 nucleotides upstream and 343 bases downstream. The plasmid pAHL-7 was constructed by ligating the 1,344-bp *Sal*I fragment of plasmid p87-2 (Fig. S2) into the corresponding restriction site of backbone vector pBBR1MCS-5 [70] (see Table1). The mutant BG87 was complemented with 723 bases of *tofR* plus 374 bases upstream and 170 bases downstream. A fragment of 1,267-bp containing *tofR* was obtained from the plasmid p1232 digested with *Sal*I-*Xho*I (Thermo Fisher Scientific) (Fig. S3) and cloned in the *Xho*I site of pBBR1MCS-5, yielding plasmid pQSR-15 (Fig. S4). The plasmid pQSR-15 was transformed in BG87. Both inserts were confirmed by sequencing.

**Ethical approval**: The conducted research is not related to either human or animals use.

## 3 Results and discussion

### 3.1 Screening of antimicrobial activity impaired mutants

We employed random mutagenesis for identifying *loci* associated with antimicrobial activity of *B. gladioli* UAPS07070. A library of 3,500 random mutants of *B. gladioli* UAPS07070 was analyzed. To show antimicrobial activity to *B. gladioli* UAPS07070, the gram-negative strain *Acinetobacter* sp. UAPS0169 was used. This strain exhibits fast and homogenous growth that allows for the clear detection of inhibition halos. The mutants, BG1232, BG87 and BG79 exhibited remarkable reduction of antimicrobial activity against *Acinetobacter* sp. UAPS0169 (Fig. 1B). The *Himar1* insertions in BG1232 and in BG87 were located close to each other allowing for the ligation of a 2,098 bp DNA segment (Fig. 2A). The sequence showed high similarity to *tofI*, *tofM* and *tofR* sequences (Table 2), which is related to the Acyl homoserine lactone quorum sensing system (AHL QS). The insertion in BG1232 interrupted a sequence corresponding to the putative Acyl-homoserine lactone synthase *tofI* (Table 2), and in the mutant BG87, affected the putative QS regulator *tofR* (Table 2, Fig. 2A). The QS circuitry that regulates pathogenicity of both *B. gladioli*, including UAPS07070 and *B. glumae* corresponds to the topological arrangement of M1 described in Proteobacteria [71,72]. The arrangement in the group M1 is characterized by the divergent transcription of *tofI* and *tofR*. Besides, *tofM*, a negative regulator homolog to *rsaM*, is found in the intergenic region between *tofI* and *tofR*. Both *tofM* and *tofI* are transcribed in tandem, and *tofR* is transcribed divergently to *tofMI* [72]. Domain search of TofI of *B. gladioli* UAPS07070 with Interproscan identified domains related to acyl transferase activity, autoinductor synthase activity and autoinductor binding (Fig. S5). The same analysis of TofR showed the presence of four characteristic domains of LuxR (Fig. S6).

**Fig. 1:**
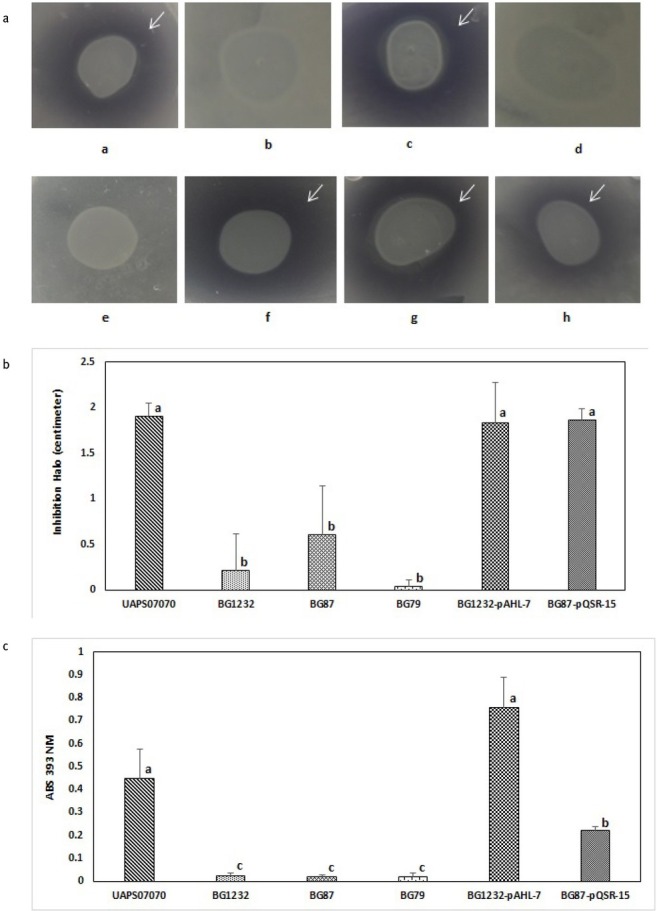
**Role of Quorum-sensing and LysR in antagonism, toxoflavin synthesis, motility, and virulence. A**, Acyl homoserine lactones detection. **a**, UAPS07070; **b**, BG1232; **c**, BG1232-pAHL-7; **d**, BG1232-pBBR1MCS-5; **e**, CV026; **f**, BG87; **g**, BG87-pQSR-15; **h**, BG87-pBBR1MCS-5. Positive reaction is by the presence of a violet halo around of the colony (marked with a white arrow). An aliquot of 10 μl of the suspension of the tested cells was placed onto a dry layer of *C. violaceum* CV026 distributed over a PSUC plate. **B**, Antagonism assay against *Acinetobacter* sp. UAPS0169. Inhibition halos produced by UAPS07070, BG1232, BG87, BG79, BG1232-pAHL-7 and BG87-pQSR-15. Double layer assay in LB plates incubated for 24 h. S.D. Standard deviation of five replicates. **C**, Toxoflavin detection. Toxoflavin level detected to 393 nm of absorbance with a standard deviation of six repeats of the strain UAPS07070, BG1232, BG87, BG79, BG1232-pAHL-7 and BG87-pQSR-15. **D**, Motility assay. **a** and **e**, UAPS07070; **b**, BG1232; **c**, BG1232-pAHL-7; **d**, BG1232-pBBR1MCS-5; **f**, BG87; **g**, BG87-pQSR-15; **h**, BG87-pBBR1MCS-5. Motility was determined in LB agar, 0.4 %, after incubation for 48 h at either 30 or 37°C. **E** Virulence lettuce assay, the inoculated strains are indicated in the figure. Observations was determined after incubation for 5 days at 30°C. The arrows indicate control inoculation.

**Fig. 1:**
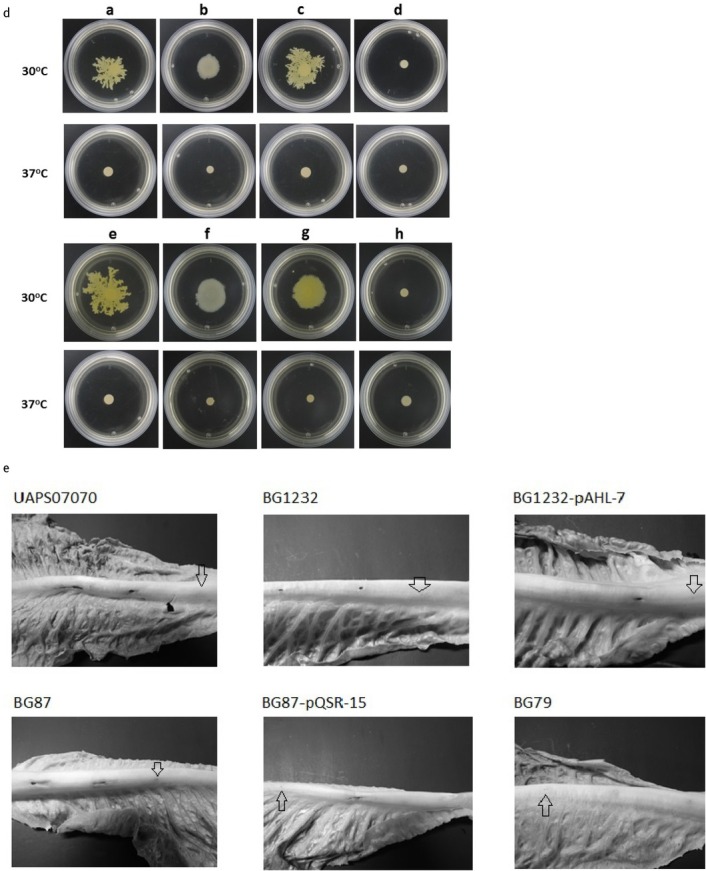
**Role of Quorum-sensing and LysR in antagonism, toxoflavin synthesis, motility, and virulence. A**, Acyl homoserine lactones detection. **a**, UAPS07070; **b**, BG1232; **c**, BG1232-pAHL-7; **d**, BG1232-pBBR1MCS-5; **e**, CV026; **f**, BG87; **g**, BG87-pQSR-15; **h**, BG87-pBBR1MCS-5. Positive reaction is by the presence of a violet halo around of the colony (marked with a white arrow). An aliquot of 10 μl of the suspension of the tested cells was placed onto a dry layer of *C. violaceum* CV026 distributed over a PSUC plate. **B**, Antagonism assay against *Acinetobacter* sp. UAPS0169. Inhibition halos produced by UAPS07070, BG1232, BG87, BG79, BG1232-pAHL-7 and BG87-pQSR-15. Double layer assay in LB plates incubated for 24 h. S.D. Standard deviation of five replicates. **C**, Toxoflavin detection. Toxoflavin level detected to 393 nm of absorbance with a standard deviation of six repeats of the strain UAPS07070, BG1232, BG87, BG79, BG1232-pAHL-7 and BG87-pQSR-15. **D**, Motility assay. **a** and **e**, UAPS07070; **b**, BG1232; **c**, BG1232-pAHL-7; **d**, BG1232-pBBR1MCS-5; **f**, BG87; **g**, BG87-pQSR-15; **h**, BG87-pBBR1MCS-5. Motility was determined in LB agar, 0.4 %, after incubation for 48 h at either 30 or 37°C. **E** Virulence lettuce assay, the inoculated strains are indicated in the figure. Observations was determined after incubation for 5 days at 30°C. The arrows indicate control inoculation.

**Table 2:** Identity of the QS and *lysR* peptides identified in *B. gladioli* UAPS07070.

	TofI	TofM	TofR	LysR
***B. gladioli* BSR3**	**98.5%**	**97.3%**	**100%**	**99.4%**
	AEA63554.1	AEA63555.1	AEA63556.1	AEA63716.1
***B. glumae* BGR1**	**86.7%**	**78.7%**	**92.1%**	**83.5%**
	ACR31808.1	ACR31807.1	ACR31806.1	ACR31745.1
***B. glumae* PGI**	**86.2%**	**79.3%**	**94.6%**	**87.4%**
	AJK49063.1.1	AJK49064.1	AJK49065.1	AJK49176.1

The identity was determined by ClustalW alignment of the amino acids.

**Fig. 2:**
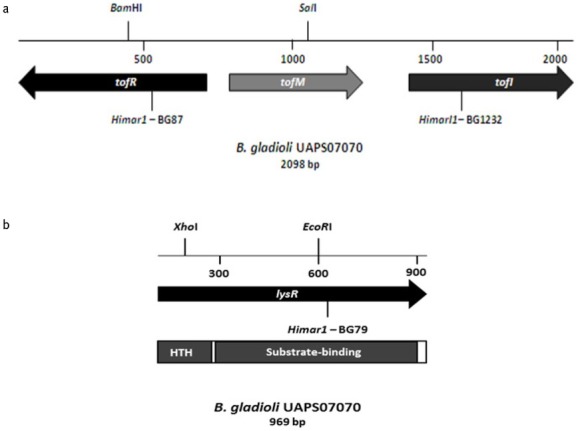
**A**, Map of the quorum sensing region of *B*. *gladioli* UAPS07070. The site of insertion of the transposon is indicated with ‘*Himar1* followed by the mutant name’. **B**, Map of the *lysR* gene region of *B*. *gladioli* UAPS07070. The site of the insertion of the transposon is indicated with ‘*Himar1* followed by the mutant name’. The principal domains of LysR-type protein, DNA binding domain (HTH) and co-inducer binding domain (substrate binding) in the LysR-type protein of UASP07070 are indicated below.

The *tofIMR* genes are implicated in the regulation of toxoflavin biosynthesis and swarming movement in the plant-pathogenic bacteria, *B. glumae* BGR1, 336gr-1 and *B. gladioli* BSR3 [49, 50, 51, 52, 53,,73]. Toxoflavin is a virulence factor demonstrated by the phytopathogen *B. glumae* [49,50,74,75] and potentially involved in the phytopathogenicity of *B. gladioli* [53]. In both models, *N-*hexanoyl homoserine lactone (C6-HSL), and *N-*octanoyl homoserine lactone (C8-HSL), are synthesized by TofI, the ortholog of the AHL synthase LuxI [49,76]. This TofR-C8-HSL complex activates the regulatory cascade of toxoflavin synthesis and its transport in *B. glumae* [49]. Besides its virulence role in plant-pathogenic bacteria, toxoflavin shows strong antimicrobial activity. Recently, the genome analysis of 88 isolates of a *B. gladioli* collection revealed that the toxoflavin biosynthetic pathway is conserved across all genomes [77]. The genetic element interrupted by the transposon *Himar1* in BG79 shows a 99% nucleotide identity with a gene of the *lysR* family, harbored in chromosome 2 of *B. gladioli* BSR3, nt 1581223 to nt 1582191 (GenBank CP002600.1). The gene is transcribed independently and encodes for a LysR-type protein of 322 a.a. (Table 2). The *Himar1* insertion was located near the C-terminal domain (nucleotide 634) that is essential for binding the co-inducer (Fig. 2B). The antibiosis of BG79 against the sensitive strain was severely diminished, suggesting a positive regulation of genetic elements involved in the synthesis and/or transport of toxoflavin. All of the *B. gladioli* sequenced genomes, including BSR3, show this same region with identical gene context among them (Results not shown). In all of them, downstream to the *lysR* locus there is a hypothetical protein in the opposite transcription direction. Upstream to *lysR* there is one hypothetical OmpC family outer membrane protein and one hypothetical protein, both in the opposite transcription direction to the *lysR* gene. In the regulatory circuit of toxoflavin production by *B. glumae* BGR1, ToxR positively regulates the toxoflavin biosynthesis and transport genes. It is a LysR-type protein that recognizes toxoflavin as a co-inducer [49]. The sequence of *lysR*, is interrupted by *Himar1* in BG79, and exhibits low sequence homology to the *toxR* sequences of *B. glumae* BGR1 and *B. gladioli* BSR3 (data not shown) suggesting that it could detect a toxoflavin alternative co-inducer.

We explored the production of a yellowish pigment, indicative of toxoflavin production [49] in *B. gladioli* UAPS07070. Normally, UAPS07070 produces toxoflavin on solid medium. However, the mutants BG1232, BG87, and BG79 do not (Fig. 1C). It has been reported that some *B. gladioli* phytopathogenic genotypes produces toxoflavin, and it is QS-dependent [52,53]. *B. gladioli* UAPS07070 is an endophytic bacterium isolated from inner tissues of pineapple. It exhibits strong antimicrobial activity against several bacteria and fungi [39]. It might also antagonize the phytopathogenic bacterium *Tatumella ptyseos* under natural conditions in pineapple plants [39].

While our results indicate that UAPS07070 synthesizes toxoflavin in culture media (Fig. 1C). We cannot discount the production of other antimicrobial metabolites. Kim *et al*. [52] observed that the expression of *B. gladioli* BSR3

polyketide genes related to the synthesis of the putative antibiotic bacillaene is QS-dependent [52]. Furthermore, other genotypes of *B. gladioli* synthesize antagonistic molecules like enacyloxin, bongkrekic acid, gladiolin, and a cyclic peptolide antibiotic [38,40,41,48,77], but the relationship to QS regulation remains to be elucidated. In addition to QS, the regulation of the toxoflavin synthesis in *B. glumae* includes an alternative pathway comprised of different *loci*, among them an orphan *luxR* [50,51,78]. The *B. gladioli* mutants BG87 and BG1232 exhibit slight antimicrobial activity against the sensitive strain *Acinetobacter* sp. UAPS0169 (Fig. 1B). This suggests alternative pathways of regulation in the residual production of inhibitory substances in UAPS07070 or the production of multiple antimicrobial molecules, as has been reported in *Pantoea agglomerans* Eh318 or *Pseudomonas protegens* Pf-5 [79,80].

### 3.2 Search of putative lux-box and autoinducer signal detection

The mutated region was aligned with the cep-box consensus sequence of *B. cenopacia* and *B. ambifaria* [54,81]. These two lux-boxes are well characterized in species of *Burkholderia*. A putative box was detected between positions 63-80 nucleotides upstream of the first ATG in *tofI* (Table S1). While *tofM* exhibits a putative lux-box 36 bp upstream of the start codon (Table S1). There was no lux-box found upstream of *tofR* (Table S1). We also did not find any lux-box upstream to *lysR* gene of BG79 (data not shown). The synthesis of signaling molecules associated to the putative QS system of UAPS07070 was confirmed with the reporter strain *C. violaceum* CV026. Apparently BG1232 did not synthesize short chain N-acyl homoserine lactones (Fig. 1A). In *B. glumae*, TofR, the ortholog to LuxR, activates the transcription of itself and of *tofI* in the presence of C8-HSL [52]. Interestingly, the mutant in the regulator, *B. gladioli* BG87, was not affected in the synthesis of signal molecules detected by *C. violaceum* CV026 (Fig. 1A). In that mutant the transposon insertion is close to the codon of residue D70 of TofR (TraR nomenclature of *Agrobacterium tumefaciens*), which is essential for the activity of this regulator [82,83]. In *B. glumae* BGR1, the expression of *tofI* is completely dependent on the TofR-C8-HSL complex [49]. Thus, a *tofR* mutant derived of *B. glumae* 336gr-1 does not synthesize AHLs [50]. In contrast, a different regulation of *tofI* is exhibited by *B. gladioli* UAPS07070. The mutant BG87 (*tofR*::*Himar1*) still synthesized AHLs, demonstrating that

UAPS07070 *tofI* can be transcribed regardless of the *tofR* mutation. An example of TofR-independent regulation of *tofI* is demonstrated by *B. pseudomallei*, in which two of three AHL synthases (*bpsI2* and *bpsI3*) are expressed constitutively [84]. In UAPS07070 the constitutive expression of *tofI* might provoke a fast response to environmental changes before reaching high cell density. This aligns with the production of inhibitory molecules by UAPS07070 in the exponential phase [39]. Another possible explanation is that *tofI* might possess another regulatory sequence besides the lux box. Further research is required to clarify the self-regulation QS system in *B. gladioli* UAPS07070.

### 3.3 Detection of *toxA* expression

**Fig. 3:**
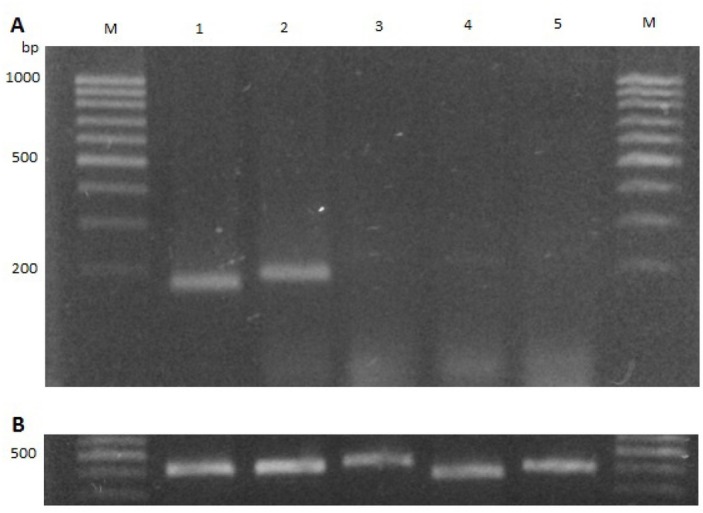
End-point RT-PCR of *toxA* gene. **A**, Agarose gel electrophoresis of *toxA* amplified by approximately 181 bp: lane 1 UAPS07070 (gDNA); lane 2 UAPS07070; lane 3 BG1232; lane 4 BG87; lane 5 BG79, line M leader 100 bp (GeneCraft). **B**, End-point RT-PCR of 16S rDNA gene of approximately 412 bp: lane 1 UAPS07070 (gDNA); lane 2 UAPS07070; lane3 BG1232; lane 4 BG87; lane 5 BG79.

The wild type strain, *B. gladioli* UAPS07070, released a deep yellowish pigment into the agar which is indicative of toxoflavin synthesis. This in contrast to the mutants BG1232, BG87 and BG79 (Fig. 1C). Two of the genes for the synthesis of toxoflavin, *toxA* and *toxB* in *B. gladioli* UAPS07070 were sequenced and compared by BLAST (data not shown). The transcription of *toxA* under experimental condition was evaluated using end-point RT-PCR. This revealed undetectable transcription of *toxA* in the mutants BG1232, BG87 and BG79 (Fig. 3). In contrast, an intense amplicon of *toxA* from RNA was observed in *B. gladioli* UAPS07070 (Fig. 3). These results strongly suggest toxoflavin acting as an antimicrobial of *B. gladioli* UAPS07070 here.

The transcription of *toxA* was undetectable in BG79 indicating a regulatory role of the LysR-type protein for the synthesis of toxoflavin. Our data revealed the participation of QS and of a LysR-type regulator in the toxoflavin production. However, the dependency between both regulation systems is unknown. To elucidate if both regulation systems are related, we explored the synthesis of acyl homoserine lactone by BG79. We found that the synthesis of autoinducer molecules was not hindered in BG79 (Fig. 1A), suggesting a higher hierarchical position of the system QS than the regulator LysR-type.

### 3.4 Swarming phenotype and virulence in onion and lettuce

Swarming is a multicellular movement over a solid or semisolid surface, and requires flagellae, cell-cell interactions and surfactant [55]. This is exhibited by *B. gladioli* UAPS07070 at 30°C with a dendritic pattern (Fig. 1D). A previous study in *B. gladioli* BSR3 showed QS-dependent swarming motility at 28°C with a movement pattern different than the dendritic one [52]. The interruption of *tofI* or *tofR* genes altered the swarming phenotype of *B. gladioli* UAPS07070 at 30°C (Fig. 1D). This may be related to the QS regulation of the synthesis of rhamnolipids, as what occurs in *B. glumae* [58]. In that model, a *tofI* mutant showed a significantly diminished synthesis of surfactant and decreased swarming activity. We show clearly that in *B. gladioli* UAPS07070, swarming motility is QS-dependent.

*B. gladioli* UAPS07070 inhabits pineapple as an avirulent bacterium [85]. In *in vitro* assays, we detected synthesis of toxoflavin, an important virulence factor involved in the pathogenicity of *B. gladioli* and *B. glumae* in rice and onion [53,75]. Although we have not detected pathogenic activity of *B. gladioli* UAPS07070 in pineapple. In *in vitro* assays in onions, this strain induces pathogenic symptoms. Peculiarly, the BG1232, BG87 and BG79 mutants seem to induce tissue maceration in onion bulb scales (Results not shown). Mutants BG1232 and BG79 showed less severe soft rotting in lettuce leaves (Fig. 1E). On the other hand, mutant in the *tofR* locus, BG87, did not show apparent decrease in virulence in lettuce (Fig. 1E). This is probably due to the AHL synthesis that could regulate toxoflavin-independent virulence mechanisms (Fig. 1A). QS regulatory networks of *B. glumae*, the close relative to *B. gladioli* are diverse as has been shown for strains BGR1 and PG1 [49,86]. Pathogenicity in *B. glumae* PG1 seem to be regulated by two different quorum sensing systems while in BGR1 is only regulated by one system [49,86]. *B. gladioli* BSR3 possess two distant quorum systems in different replicons (Acc. Nums. NC_015376 and NC_015382) [72]. It is unknown if UAPS07070 possesses more than one quorum loci and if one or both of them are related to pathogenicity. Lee *et al*. [53] reported that between *B. gladioli* and *B. glumae* there are differences in the participation of quorum sensing regulation. *B. gladioli* strain KACC11889 carries the genetic elements for the biosynthesis of toxoflavin but does not possess the quorum sensing genes for activating the synthesis of the phytotoxin. Nevertheless, *B. gladioli* KACC11889 still induces tissue maceration of onion bulb scales and exhibits swarming movement and cannot cause damage in rice. Thus, the regulation in this bacterium might have evolved differently to *B. gladioli* BSR3 and *B. glumae* BGR1, since in those strains the inactivation of QS system abolished the virulence in onion, toxoflavin production and the swarming motility [49,52,53,58]. Interestingly, two strains of the same species *B. glumae*, BGR1 and PG1, show different regulatory QS behavior [49,86]. It is known that *B. glumae* 237-5, an avirulent strain in rice does not produce toxoflavin and keeps its virulence in onion, indicating that the tissue maceration in onion is not attributed exclusively to toxoflavin [75].
