# Supplementary material for: Loci Identification of a N-acyl Homoserine Lactone Type Quorum Sensing System and a New LysR-type Transcriptional Regulator Associated with Antimicrobial Activity and Swarming in Burkholderia Gladioli UAPS07070
Source: Open Life Sci. 2019 Jun 24;14:165–78. doi: 10.1515/biol-2019-0019 (PMC7874821; doi:10.1515/biol-2019-0019)

Supporting Info

Table S1. Putative lux-box of *B. gladioli* UAPS07070.

*tofI*_UAPS07070	-80	CTGTCAGGAATAACAGTT	This study
*tofM*_UAPS07070	-36	CTGTCATTCTTGCTAGGT	This study
cepI box consensus[a]		CTGTAAAAGTTACCAGTT	[78]
cepI box consensus[b]		CTGTAAGAGTTGTCAGTT	[53]

[a] *B. cenocepacia*; [b] *B. ambifaria*

Fig. S1 Plasmid pAHL-7. It was constructed with a fragment of 1344-bp obtained by digestion of p87-2 with *Sal*I. This fragment contained the gen *tofI* of *B. gladioli* UAPS07070 (blue arrow) plus 386-bp upstream, harboring partially the gene *tofM* (clear blue arrow), and additionally 343-bp downstream *tofI*. The fragment *Sal*I was cloned in the *Sal*I site of pBBR1MCS-5 (Kovach et al. 1995)

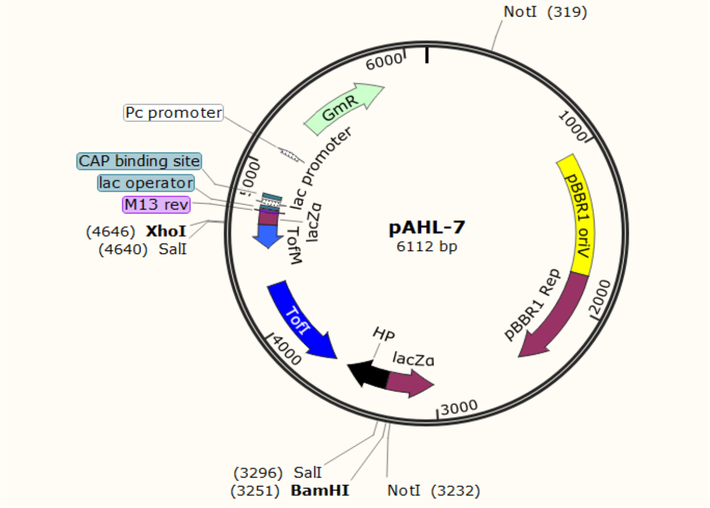

Fig. S2 Plasmid p87-2. It was obtained with the *Not*I fragment of BG87 containing the transposon plus *B. gladioli* UAPS07070 sequences. The plasmid harbors six genes of UAPS07070, three unknown genes (black arrow), the gene *tofI* (blue arrow), the gene *tofM* (clear blue arrow) and the gene *tofR* (green arrow) interrupted with the transposon *Himar1*

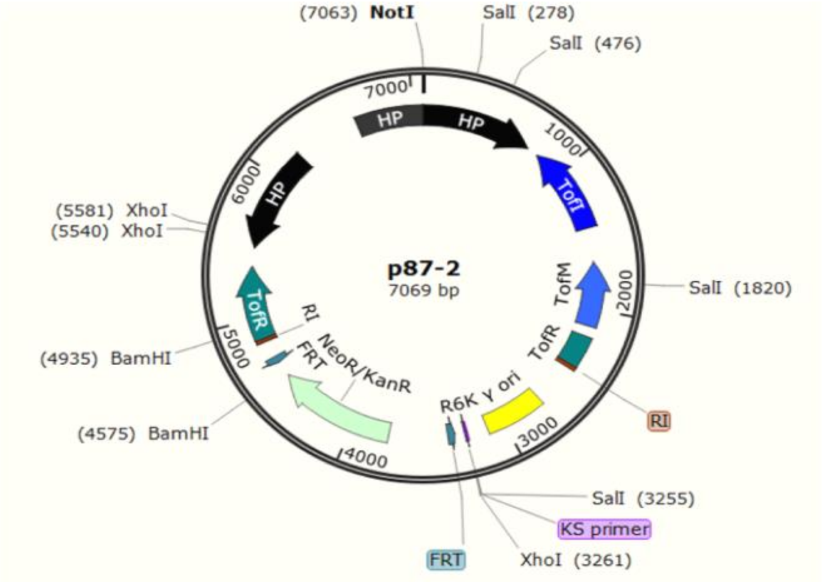

Fig. S3 Plasmid p1232. It was obtained with a *Not*I fragment of BG1232 containing the transposon plus *B. gladioli* UAPS07070 sequences. p1232 harbors six genes of UAPS07070, three unknown genes (black arrow), the *tofR* gene (green arrow), the *tofM* gene (clear blue arrow) and the *tofI* gene (blue arrow) interrupted with the transposon *Himar1*

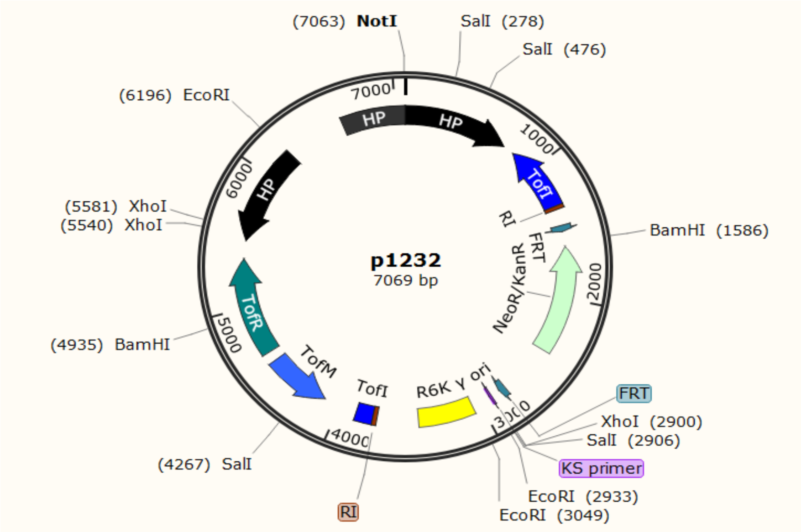

Fig. S4 Map pQSR-15 plasmid, the plasmid size is 6035-bp. pQSR-15 was constructed with 1267-bp digested with *Xho*I-*Sal*I of p1232 plasmid, this fragment contained the gen *tofR* of B. gladioli UAPS07070 (green arrow) more 343-bp upstream harboring partial *tofM* gene (clear blue arrow) and 170-bp downstream that harboring to unknown gene. The fragment *Xho*I-*Sal*I was cloned in *Sal*I site of pBBR1MCS-5 (Kovach et al. 1995). Restriction sites *Not*I, *Sal*I *and Xho*I are shown

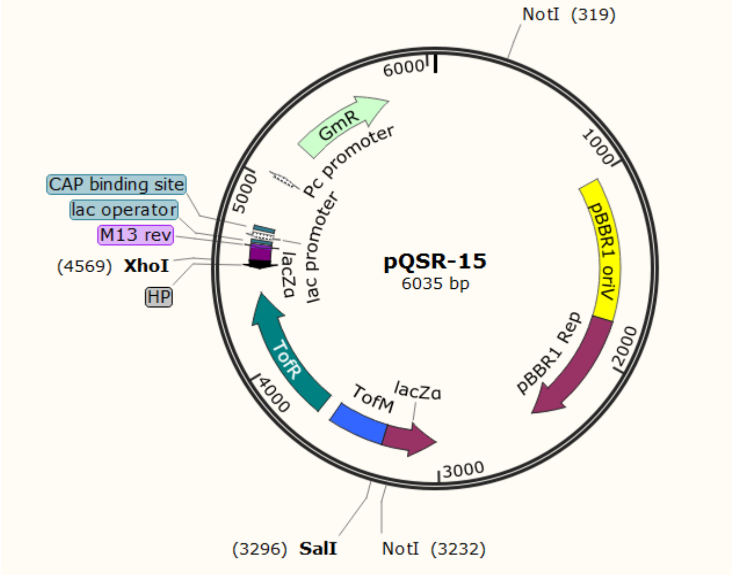

Fig. S5. Putative TofI Protein analysis in Interproscan platform: A, protein domains found in putative TofI; B, amino acid conserved in TofI protein of *B. gladioli* UAPS07070 in comparison with TraI of *Agrobacterium tumefaciens*.

A

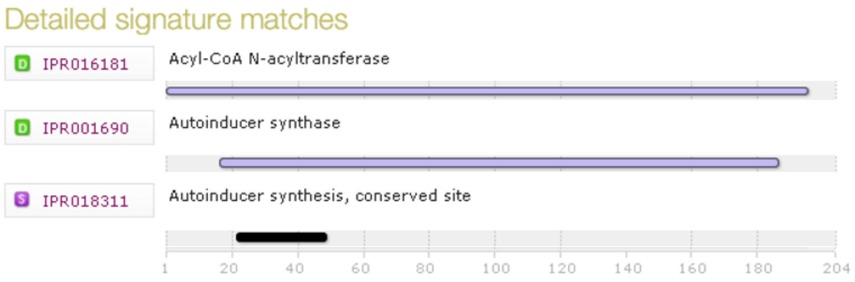

B

**TofI**	**R23**	**F27**	**W33**	**E43**	**D45**	**D48**	**R69**	**F83**	**E101**	**E104**
**TraI**	R25	F29	W35	E44	D46	D49	R70	F84	E101	E104

Fig. S6. Putative TofR Protein analysis in Interproscan platform: A, protein domains found in putative TofR; B, amino acid conserved in TofR protein of *B. gladioli* UAPS07070 in comparison with TraR of *Agrobacterium tumefaciens*.

A

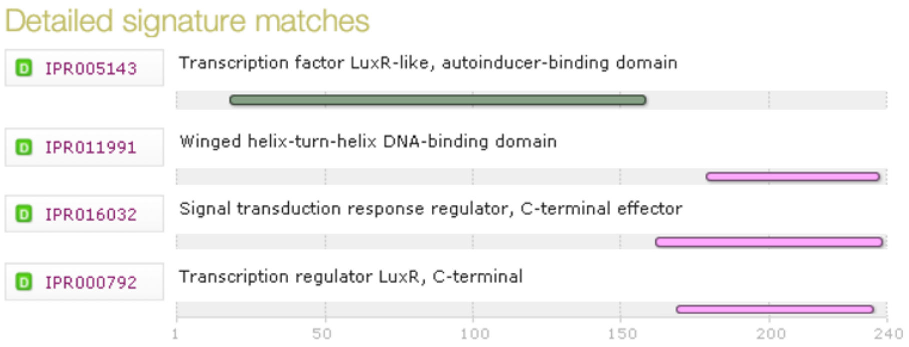

B

**TofR**	**W63**	**Y67**	**D76**	**P77**	**W91**	**G115**	**E181**	**L185**	**G191**
**TraR**	W57	Y61	D70	P71	W85	G113	E178	L182	G188

Fig. S7. p87-2. Plasmid of 7069 pb obtained by recircularizing a *Not*I fragment of the mutant BG87 containing the transposon *Himar1* plus *B. gladioli* UAPS07070 sequences: HP, unknown genes codifying for hypothetical protein (black arrow);TofI, gene *tofI* (blue arrow); TofM, gene *tofM* (clear blue arrow); TofR, gene *tofR* (green arrow); NeoR/KanR, cassette Kanamycin resistance; RI, inverted repeat; FRT, Flp recombinase targets; R6K, π protein-dependent R6K replication origin; Restriction site for *Not*I, *Sal*I, *BamH*I, and *Xho*I are indicated in the figure.

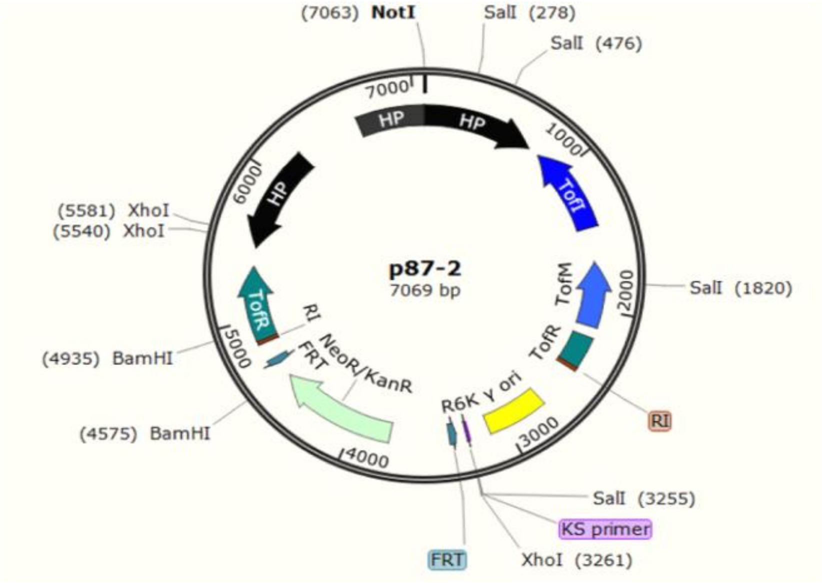

Fig. S8. p1232. Plasmid of 7069 bp obtained by recircularizing a *Not*I fragment of the mutant BG1232 containing the transposon *Himar1* plus *B. gladioli* UAPS07070 sequences: HP, unknown genes codifying for hypothetical protein (black arrow);TofI, gene *tofI* (blue arrow); TofM, gene *tofM* (clear blue arrow); TofR, gene *tofR* (green arrow); NeoR/KanR, cassette Kanamycin resistance; RI, inverted repeat; FRT, Flp recombinase targets; R6K, π protein-dependent R6K replication origin; Restriction site for *Not*I, *Sal*I, *BamH*I, *EcoR*I, and *Xho*I are indicated in the figure.

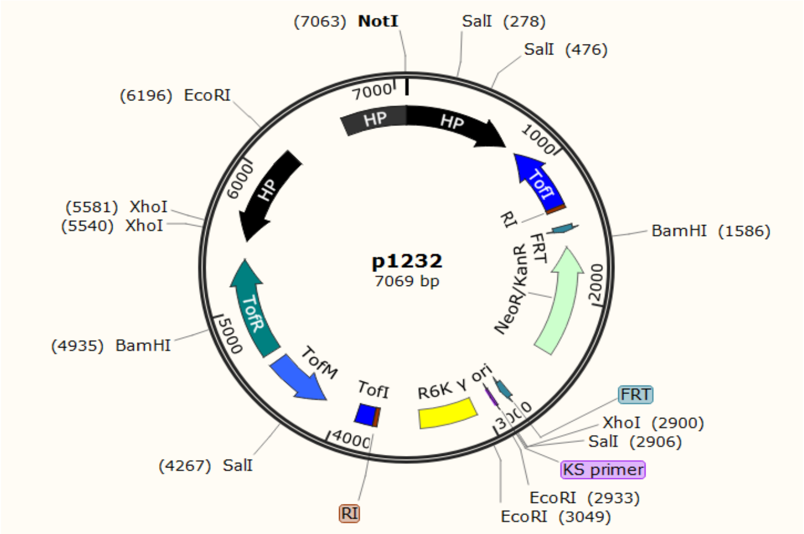

Fig. S9. P79-1. Plasmid of 10,437 bp obtained by recircularizing a *Not*I fragment of the mutant BG79 containing the transposon *Himar1* plus *B. gladioli* UAPS07070 sequences: HP, unknown genes codifying for hypothetical protein (black arrow); LysR, gene *lysR* (orange arrow; NeoR/KanR, cassette Kanamycin resistance; RI, inverted repeat; FRT, Flp recombinase targets; R6K, π protein-dependent R6K replication origin; Restriction site for *Not*I and *Sal*I, are indicated in the figure.

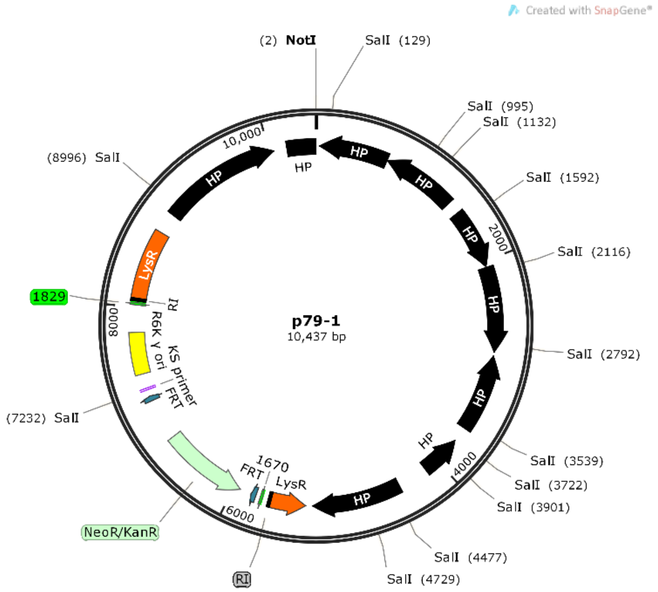

Supplement: Supplementary file 1 [file biol-14-165_sm.pdf]
